# Quantization and Bifurcation beyond Square-Integrable Wavefunctions

**DOI:** 10.3390/e20050327

**Published:** 2018-04-29

**Authors:** Ciann-Dong Yang, Chung-Hsuan Kuo

**Affiliations:** Department of Aeronautics and Astronautics, National Cheng Kung University, No. 1, University Road, Tainan 701, Taiwan

**Keywords:** square integrable, energy quantization, Quantum Hamilton-Jacobi Formalism, quantum trajectory

## Abstract

Probability interpretation is the cornerstone of standard quantum mechanics. To ensure the validity of the probability interpretation, wavefunctions have to satisfy the square-integrable (SI) condition, which gives rise to the well-known phenomenon of energy quantization in confined quantum systems. On the other hand, nonsquare-integrable (NSI) solutions to the Schrödinger equation are usually ruled out and have long been believed to be irrelevant to energy quantization. This paper proposes a quantum-trajectory approach to energy quantization by releasing the SI condition and considering both SI and NSI solutions to the Schrödinger equation. Contrary to our common belief, we find that both SI and NSI wavefunctions contribute to energy quantization. SI wavefunctions help to locate the bifurcation points at which energy has a step jump, while NSI wavefunctions form the flat parts of the stair-like distribution of the quantized energies. The consideration of NSI wavefunctions furthermore reveals a new quantum phenomenon regarding the synchronicity between the energy quantization process and the center-saddle bifurcation process.

## 1. Introduction

In the statistical formulation of quantum mechanics, a wavefunction ψ has to be square integrable (SI) to ensure the qualification of $\psi^{*}\psi$ as a probability density function. SI solutions to the Schrödinger equation can be used to determine the energy levels in a confined system. Nonsquare-integrable (NSI) solutions to the Schrödinger equation otherwise are ruled out and their role has been unknown till now. To investigate the role of NSI wavefunctions, we need a formulation of quantum mechanics, which does not require the SI condition. Among the nine different formulations of quantum mechanics [1], there is a formulation known as the quantum Hamilton-Jacobi (H-J) formalism [2,3], which meets our purpose. The quantum H-J formalism has been developed since the inception of quantum mechanics along the line of Jordan [4], Dirac [5] and Schwinger [6]. The main advantage of the classical H-J formalism is to give the frequencies of a periodic motion directly without solving the equations of motion. Analogous to its classical counterpart, the advantage of quantum H-J formalism is recognized as a method of finding energy eigenvalues directly without solving the related Schrödinger equation.

Based on the quantum H-J equation, Leacock and Padgett [2,3] proposed an ingenious method to evaluate energy eigenvalues by contour integral. This approach to energy eigenvalues $E_{n}$ is entirely independent of whether the related wavefunction is SI or not, and allows us to examine the participation of the NSI wavefunctions in the process of energy quantization. Apart from providing energy eigenvalues, quantum H-J formalism like its classical counterpart produces quantum Hamilton dynamics [7], from which complex quantum trajectories can be solved to describe the quantum motion associated with a given wavefunction. Probability interpretation isolates SI wavefunctions from NSI wavefunctions; on the contrary, under the quantum H-J formalism SI and NSI wavefunctions are indivisible with continuously connected quantum trajectories. Because NSI wavefunctions ψ fail to serve as probability density functions, we need an alternative operation to replace the expectation (assemble average) $\langle \psi |\hat{\Omega }|\psi \rangle$ of a quantum observable Ω. The complex quantum trajectory method developed from the quantum H-J formalism can provide the time average ${\langle \Omega \rangle }_{T}$ to substitute for the usual assemble average $\langle \psi |\hat{\Omega }|\psi \rangle$.

Based on the time-average operation ${\langle \Omega \rangle }_{T}$, which applies to both SI and NSI wavefunctions, we can derive quantization laws more general than those based on the assemble average $\langle \psi |\hat{\Omega }|\psi \rangle$, which applies only to SI wavefunctions. One of the general results shows that as the total energy E of a confined system increases monotonically, the time-average kinetic energy ${\langle E_{k}\rangle }_{T}$ of a confined particle exhibits a stair-like distribution in such a way that the step jumps occur as E equal to one of the energy eigenvalue $E_{n}$ and the flat part of the distribution is formed over the interval $E_{n}\leq E<E_{n+1}$. During the process as E increases continuously from $E_{n}$ to the next energy eigenvalue $E_{n+1}$, we note that all the corresponding wavefunctions are NSI, but they all yield the same value of ${\langle E_{k}\rangle }_{T}$ and form the flat part of the stair-like energy distribution. In other words, the transition from the eigenstate $\psi_{n}$ to the next eigenstate $\psi_{n+1}$ can be connected smoothly by the NSI wavefunctions $\psi_{E}$ with $E_{n}<E<E_{n+1}$, which otherwise have been ruled out in standard quantum mechanics.

Compared to the complex quantum trajectory derived from the quantum H-J formalism, de Broglie-Bohm (dBB) quantum trajectory [8,9,10] is real-valued. The equivalence between dBB trajectory interpretation and probability interpretation of quantum mechanics has been well developed over the last several decades. Although under the dBB formulation, particles follow continuous trajectories with well-defined two-time position correlations, a recent paper by Gisin [11] pointed out that Bohmian mechanics makes the same predictions as standard quantum mechanics: the violation of Bell inequalities. The studies of dBB formulation of quantum mechanics [12,13,14] revealed that like the way that thermal probabilities arise in ordinary statistical mechanics, the quantum probabilities ${\left|\psi \left(x,t\right)\right|}^{2}$ arise dynamically in a similar way that a simple initial ensemble with a non-equilibrium distribution $P\left(x,0\right)\neq {\left|\psi \left(x,0\right)\right|}^{2}$ of particle positions evolves towards the equilibrium distribution via the relaxation process $P\left(x,t\right)\to {\left|\psi \left(x,t\right)\right|}^{2}$. Meanwhile, the speed of the convergence of P(x,t) to ${\left|\psi \left(x,t\right)\right|}^{2}$ was found to correlate with the degree of chaos of the involved Bohmian trajectories [15,16,17], which in turn was shown to be related to the vortex dynamics generated by nodal points in the wavefunction ψ(x,t) [18,19]. The degree of chaos produced by many interacting vortices ultimately depends on the number and spatial distribution of the nodal points in the configuration space [20,21].

Parallel to the development of real-valued dBB trajectories, the study of complex-valued quantum trajectories has evolved into a quantum trajectory method, which integrates the hydrodynamic equations *on the fly* to synthesize the probability density by evolving ensembles of complex quantum trajectories [22,23,24]. Due to the additional degree of freedom given by the imaginary part of a complex trajectory, it is possible to synthesize the quantum probability ${\left|\psi \left(x,t\right)\right|}^{2}$ by a single complex-valued trajectory, instead of an ensemble of real-valued or complex-valued trajectories [25].

To date, the trajectory approaches to quantum mechanics, either using real-valued trajectories based on dBB formulation or using complex-valued trajectories based on H-J formulation, mainly deal with SI wavefunctions in order to show their consistency with the probability interpretation. Here we will go beyond SI wavefunctions to find out what will happen, when statistical interpretation is not applicable. On one hand we will use complex trajectories to demonstrate the energy quantization process after releasing the SI condition, and on the other we will apply the quantum Hamilton dynamics to demonstrate the sequential center-saddle bifurcations as the energy quantization proceeds. The combined result manifests a new quantum phenomenon regarding the synchronicity between the energy quantization process and the center-saddle bifurcation process.

NSI wavefunctions not only participate in the quantization and bifurcation process, but also in the formation of spin degree of freedom. In spite of their distinct statistical properties, SI and NSI wavefunctions have similar velocity fields with the only difference in their directions of rotation on the complex plane. As the third goal of the paper, we will contrast quantum trajectories of SI wavefunctions with those of NSI wavefunctions to manifest the invisible spin degree of freedom as a rotational motion on the complex plane.

The remainder of this paper is organized as follows: Section 2 presents quantum H-J formalism and the related method of determining energy eigenvalues. In Section 3, time average operation for NSI wavefunctions along a complex quantum trajectory is developed from the quantum H-J formalism to replace the ensemble average. The proposed time average operation is then used in Section 4 to derive the universal quantization laws regarding the kinetic energy and the quantum potential. Section 5 demonstrates the participation of NSI wavefunctions in the energy quantization process for a harmonic oscillator. Section 6 proposes a quantum dynamic description of energy quantization, in terms of which a new phenomenon regarding the synchronicity between quantization and bifurcation is revealed. Finally, both SI and NSI solutions to the Schrödinger equation are considered in Section 7 and their relations to spin degree of freedom are explained.

## 2. Quantum Hamilton-Jacobi Formalism

While the quantum H-J theory is general, here we consider its application to bound states, which have quantized energy levels and closed quantum trajectories. The quantum H-J approach to determining energy eigenvalues can be conceived of as an extension of the Wilson-Sommerfeld quantization rule [26]. In this approach, the quantum energy levels are given exactly by setting the quantum action variable equal to an integer multiple of Planck constant:

(1) $$J\left(E\right)=\frac{1}{2\pi }\oint_{c}^{}p\left(x\right)dx=n\hbar ,\;n=0,\;1,\;2,\;\cdots ,$$

where p(x) is called quantum momentum function (QMF) and the contour c is defined on the complex plane with the integer n being the number of poles of p(x) enclosed by c. The QMF p(x) is related to the quantum action function S and the wavefunction ψ as:

(2) $$p\left(x\right)=\frac{\partial S}{\partial x}=-i\hbar \frac{\partial \ln\psi }{\partial x},$$

with S satisfying the quantum H-J equation:

(3) $$\frac{\partial S}{\partial t}+{H\left(t,x,p\right)|}_{p=\partial S/\partial x}=\frac{\partial S}{\partial t}+{\left[\frac{p^{2}}{2m}+V-\frac{i\hbar }{2m}\frac{\partial p}{\partial x}\right]}_{p=\partial S/\partial x}=0,\;$$

and with ψ satisfying the Schrödinger equation:

(4) $$i\hbar \frac{\partial \psi }{\partial t}=-\frac{\hbar^{2}}{2m}\frac{\partial^{2}\psi }{\partial x^{2}}+V\psi .\;$$

It appears that the quantum H-J Equation (3) and the Schrödinger Equation (4) are equivalent expressions via the relation S=−iℏlnψ.

Leacock and Padgett [2,3] proposed an ingenious method to evaluate J(E) without actually solving p(x) from the quantum H-J Equation (3). They showed that for a given potential V(x), J(E) can be computed simply by a suitable deformation of the complex contour c and the change of variables in Equation (1). Once J(E) is found, the energy eigenvalues $E_{n}$ can be determined by solving E in terms of the integer n via the relation J(E)=nℏ.

The quantum H-J approach to determining energy eigenvalues has two significant implications. Firstly, this approach suggests that the energy eigenvalue $E_{n}$ stems from the quantization of the action variable J, rather than from the quantization of the total energy E itself. Precisely speaking, the energy eigenvalue $E_{n}$ is the specific energy E at which the action variable J(E) happens to be an integer multiple of ℏ, i.e., $J\left(E_{n}\right)=n\hbar$. Inspired by this implication, the first goal of this paper is to reveal the internal mechanism causing the quantization of the action variable J and find out its relation to the energy quantization.

Secondly, the quantum H-J approach implies that the SI condition is not required throughout the process of determining energy eigenvalues, which means that whether wavefunctions are SI or not is unconcerned upon evaluating eigen energies. Based on this observation, our second goal here is to expose how SI and NSI wavefunctions cooperate to form the observed energy levels within a confining potential. For a given wavefunction ψ(t,x) either SI or NSI, the associated quantum dynamics can be described by the quantum Hamilton equations with the quantum Hamiltonian H given by Equation (3):

(5a) $$\frac{dx}{dt}=\frac{\partial H}{\partial p}=\frac{p}{m},\;x\in \mathbb{C},$$

(5b) $$\frac{dp}{dt}=-\frac{\partial H}{\partial x}=-\frac{\partial }{\partial x}\left(V\left(x\right)+Q\left(t,x\right)\right),\;p\in \mathbb{C},$$

where Q(x) is the complex quantum potential defined by:

(6) $$Q\left(t,x\right)=-{\frac{i\hbar }{2m}\frac{\partial p}{\partial x}|}_{p=\partial S/\partial x}=-\frac{i\hbar }{2m}\frac{\partial^{2}S}{\partial x^{2}}=-\frac{\hbar^{2}}{2m}\frac{\partial^{2}\ln\psi \left(t,x\right)}{\partial x^{2}}.\;$$

Quantum potential Q(t,x) is intrinsic to the quantum state ψ(t,x) and is independent of the externally applied potential V(x). The quantum Hamilton Equations (5) are distinct from the classical ones in two aspects: the complex nature and the state-dependent nature. The complex nature is a consequence of the fact that the canonical variables (x,p) solved from Equation (5) are, in general, complex variables. The state-dependent nature means that Equation (5) governs the quantum motion specifically in the quantum state described by ψ. The Hamilton Equations (5), which is usually regarded as the complex-extension of Bohmian mechanics, can be derived independently by the optimal stochastic control theory [27].

For a given wavefunction ψ(t,x), the complex contour c traced by x(t) can be solved from Equation (5a), along which the contour integral in Equation (1) then can be evaluated. The second Hamilton Equation (5b) is an alternative expression of the Schrödinger Equation (4) as can be shown by substituting p(t,x) from Equation (2) and Q(t,x) from Equation (6).

## 3. Time Average along a Complex Quantum Trajectory

The necessity of considering time average along a complex quantum trajectory comes from the fact that the action variable J introduced in Equation (1) is equal to the time-average kinetic energy, as will be shown below. For a particle confined by a time-independent potential V(x), we have wavefunction $\psi \left(t,x\right)=e^{-iEt/\hbar }\psi_{E}\left(x\right)$ and quantum action function $S\left(t,x\right)=-Et-i\hbar \ln\psi_{E}\left(x\right)$, with which the quantum H-J Equation (3) can be recast into the following form:

(7) $$H\left(x,p\right)=\frac{p^{2}}{2m}+V\left(x\right)+Q\left(x\right)=-\frac{\partial S}{\partial t}=E.$$

This is the energy conservation law in the quantum H-J formalism, indicating that the conserved total energy E comprises three terms: the kinetic energy $E_{k}=p^{2}/2m$, the applied potential V(x), and the quantum potential Q(x). When expressed in terms of the wavefunction $\psi_{E}\left(x\right)$, Equation (7) and Equation (4) become the time-independent Schrödinger equation:

(8) $$\frac{\hbar^{2}}{2m}\frac{d^{2}\psi_{E}}{dx^{2}}+\left(E-V\left(x\right)\right)\psi_{E}=0.$$

The energy conservation law (7) is valid for any solution $\psi_{E}\left(x\right)$ to the Schrödinger Equation (8), either SI or NSI. The Schrodinger Equation (8) has a continuum of solutions, unless it is supplemented with appropriate boundary conditions. Without loss of generality, we consider V(x) in the form of a potential well with the property V(x)→∞, as x→±∞. Due to the presence of the infinite potential, the probability of finding the particle at infinity is zero, i.e.:

(9) $$\psi_{E}\left(x\right)\to 0,\mathrm{as}\;x\to \pm \infty .$$

This boundary condition gets rid of most of the solutions to Equation (8) and selects out only a discrete set of $\psi_{E}$ and E. Consequently, it is the boundary conditions in standard quantum mechanics that actually enforce the quantization. The boundary condition (9) originates from the fundamental requirement that wavefunctions must be SI, i.e.,:

(10) $$\int_{-\infty }^{+\infty }\psi_{E}^{*}\left(x\right)\psi_{E}\left(x\right)dx<\infty ,$$

which allows the normalization of the total probability to unity. If the SI condition (10) or the boundary condition (9) is released, the total energy E will be still conserved, but no longer quantized, because the participation of NSI wavefunctions $\psi_{E}\left(x\right)$ in Equation (7) will result in an arbitrary total energy E other than $E_{n}$. However, even if the total energy E is allowed to be varied continuously, there exist intrinsic quantization laws from which the energy eigenvalue $E_{n}$ can be recovered. In other words, probability interpretation with the accompanying SI condition is not the only way to arrive at the quantization. This issue was first addressed by Leacock and Padgett [2,3] and demonstrated in detail by Bhalla [28,29].

Although NSI wavefunctions ψ fail to serve as probability density functions in the assemble average ${\langle \Omega \left(x,p\right)\rangle }_{\psi }=\langle \psi |\Omega \left(\hat{x},\hat{p}\right)|\psi \rangle$, complex quantum trajectories for NSI wavefunction still exist, along which time average of Ω(x,p) can be defined to substitute for the assemble average ${\langle \Omega \left(x,p\right)\rangle }_{\psi }$. The complex quantum trajectory describing the particle’s motion in a confined system can be solved from Equations (5a) and (2), which together with $\psi \left(t,x\right)=e^{-iEt/\hbar }\psi_{E}\left(x\right)$ gives the governing equation as:

(11) $$\frac{dx}{dt}=-\frac{i\hbar }{m}\frac{\psi_{E}^{\prime }\left(x\right)}{\psi_{E}\left(x\right)},$$

where $\psi_{E}\left(x\right)$ is a general solution to Equation (8) with given energy E. The resulting complex trajectory x(t) serves as a physical realization of the complex contour c appearing in Equation (1), and allows the contour integral to be evaluated along the particle’s path of motion.

By treating the quantum Hamiltonian H(x,p) defined in Equation (7) as a Lyapunov function, the energy conservation law dH/dt=0 implies that the autonomous nonlinear system (11) is Lyapunov stable (neutrally stable) with equilibrium points in the form of centers, irrespective of whether $\psi_{E}$ is SI or not. The trajectory solved from Equation (11) coincides with the Lyapunov contour lines defined by H(x,p)=E=constant, which are concentric curves surrounding equilibrium points.

The time average of Ω(x,p) along the particle’s trajectory x(t) is defined as:

(12) $${\langle \Omega \left(x,p\right)\rangle }_{T}=\frac{1}{T}\int_{0}^{T}\Omega \left(x\left(t\right),p\left(t\right)\right)dt,$$

where T is the period of oscillation of x(t). The quantum action variable J defined in Equation (1) is a ready example of taking time average along a complex contour. Letting c be a closed trajectory solved from Equation (11), we can rewrite the contour integral (1) in terms of the time-average kinetic energy as:

(13) $$J=\frac{1}{2\pi }\oint_{c}^{}p\left(x\right)dx=\frac{1}{2\pi m}\int_{0}^{T}p^{2}dt=\frac{2}{\omega }{\langle E_{k}\rangle }_{T},$$

where ω=2π/T is the angular frequency of the periodic motion. Therefore, the Wilson-Sommerfeld quantization law J=nℏ is simply an alternative expression of the energy quantization law ${\langle E_{k}\rangle }_{T}=n\left(\hbar \omega /2\right)$.

In general, the time average of an arbitrary function Ω(x,p) can be expressed in terms of a contour integral by using Equations (11) and (12):

(14) $${\langle \Omega \left(x,p\right)\rangle }_{T}=i\frac{m\omega }{2\pi \hbar }\oint_{c}^{}\Omega \left(x\right)\frac{\psi_{E}\left(x\right)}{\psi_{E}^{\prime }\left(x\right)}dx,$$

where c is the closed contour traced by x(t) on the complex plane, and the symbol “prime” denotes the differentiation with respect to x. Since QMF p(x) can be expressed as a function of x, we simply write Ω(x,p) as Ω(x) in the integrand. According to the residue theorem, the value of ${\langle \Omega \left(x,p\right)\rangle }_{T}$ is determined only by the poles of the integrand enclosed by the contour c and is independent of the actual form of c. We will see below that the discrete change of the number of poles in the integrant leads to the quantization of ${\langle \Omega \left(x,p\right)\rangle }_{T}$.

## 4. General Quantization Laws without SI Condition

Let $\psi_{E}\left(x\right)$ be a general solution to the Schrödinger Equation (8) with a given energy E. We can treat the time average ${\langle \Omega \left(x,p\right)\rangle }_{T}$ as a function of the total energy E by noting that ${\langle \Omega \left(x,p\right)\rangle }_{T}$ is computed by Equation (14) with wavefunction $\psi_{E}\left(x\right)$, which in turn depends on the energy E. The time average ${\langle \Omega \left(x,p\right)\rangle }_{T}$ is said to be quantized, if its value manifests a stair-like distribution as the total energy E increases monotonically. We will derive several energy quantization laws originating from such a stair-like behavior of ${\langle \Omega \left(x,p\right)\rangle }_{T}$, which are universal for all confined quantum systems. The energy quantization defined here denotes the discrete change of the considered energy, which is different from the definition in standard quantum mechanics, where energy quantization denotes the discrete energies satisfying the SI condition (10).

Firstly, we consider the quantization of the time-average kinetic energy. By substituting $\Omega \left(x,p\right)=p^{2}/2m$ into Equation (14), we obtain:

(15) $${\langle E_{k}\rangle }_{T}=\frac{1}{T}\int_{0}^{T}\frac{1}{2m}p^{2}dt=\frac{\hbar \omega }{4\pi i}\oint_{c}^{}\frac{\psi_{E}^{\prime }\left(x\right)}{\psi_{E}\left(x\right)}dx.$$

To evaluate the above contour integral, we recall a formula from the residue theorem:

(16) $$\oint_{c}^{}\frac{\Omega^{\prime }\left(x\right)}{\Omega \left(x\right)}dx=2\pi i\left(Z_{f}-P_{f}\right),$$

where $Z_{f}$ and $P_{f}$ are, respectively, the numbers of zero and pole of Ω(x) enclosed by the contour c. Using this formula in Equation (15) yields:

(17) $${\langle E_{k}\rangle }_{T}=\frac{\hbar \omega }{2}\left(Z_{\psi }-P_{\psi }\right)=\frac{\hbar \omega }{2}n_{\psi },\;$$

where the integer $n_{\psi }=Z_{\psi }-P_{\psi }$ is the difference between the numbers of zero and pole of $\psi_{E}\left(x\right)$. It appears that the time average of the particle’s kinetic energy in a confined potential is an integer multiple of ℏω/2. This is an universal quantization law independent of the confining potential V(x). Using Equation (17) in Equation (13), we recover the Wilson-Sommerfeld quantization law $J=n_{\psi }\hbar$.

The other quantized energy is the quantum potential Q. The evaluation of Equation (14) with Ω(x,p)=Q(x) gives:

(18) $${\langle Q\rangle }_{T}=\frac{1}{T}\int_{0}^{T}Qdt=\frac{\hbar }{2miT}\int_{0}^{T}\frac{dp}{dx}dt=\frac{\hbar \omega }{4\pi i}\oint_{c}^{}\frac{p^{\prime }\left(x\right)}{p\left(x\right)}dx.$$

Applying Formula (16) once again, we arrive at the second energy quantization law:

(19) $${\langle Q\rangle }_{T}=\frac{\hbar \omega }{2}\left(Z_{p}-P_{p}\right)=\frac{\hbar \omega }{2}n_{p},$$

where integer $n_{p}=Z_{p}-P_{p}$ is the difference between the numbers of zero and pole of p(x). Like the quantization of ${\langle E_{k}\rangle }_{T}$, Equation (19) reveals that the value of ${\langle Q\rangle }_{T}$ is an integer multiple of ℏω/2, irrespective of the confining potential V(x).

The Kinetic energy $E_{k}$ and the quantum potential energy Q, individually, are quantized quantities, and their combination leads to another quantization law. This can be verified from the combination of Equations (15) and (18):

(20) $${\langle E_{k}+Q\rangle }_{T}=\frac{\hbar \omega }{4\pi i}\oint_{c}^{}\left[\frac{\psi_{E}^{\prime }\left(x\right)}{\psi_{E}\left(x\right)}+\frac{p^{\prime }\left(x\right)}{p\left(x\right)}\right]dx,$$

where in the integrand can be simplified further as:

$$\frac{\psi_{E}^{\prime }\left(x\right)}{\psi_{E}\left(x\right)}+\frac{p^{\prime }\left(x\right)}{p\left(x\right)}=\frac{d}{dx}\ln\left[p\left(x\right)\psi_{E}\left(x\right)\right]=\frac{d}{dx}\ln\psi_{E}^{\prime }\left(x\right).$$

With the above simplification and the Formula (16), Equation (20) yields a new quantization law:

(21) $${\langle E_{k}+Q\rangle }_{T}=\frac{\hbar \omega }{4\pi i}\oint_{c}^{}\frac{\psi_{E}^{\prime \prime }\left(x\right)}{\psi_{E}^{\prime }\left(x\right)}dx=\frac{\hbar \omega }{2}n_{\psi^{\prime }},$$

where integer $n_{\psi^{\prime }}=Z_{\psi^{\prime }}-P_{\psi^{\prime }}$ is the difference between the numbers of zero and pole of $\psi_{E}^{\prime }\left(x\right)$.

The three integers, $n_{\psi }$, $n_{p}$ and $n_{\psi^{\prime }}$, are solely determined by the wavefunction $\psi_{E}$, which in turn is solved from Equation (8) with a prescribed energy E. As E increases, the three integers can only change discretely in response to the continuous change of E. Let $E_{0}<\cdots <E_{n-1}<E_{n}<\cdots$ be the sequence of specific energies at which the integer $n_{\psi^{\prime }}$ experiences a step jump, n−1→n. With increasing E, the value of ${\langle E_{k}+Q\rangle }_{T}$ then assumes a stair-like distribution described by:

(22) $${\langle E_{k}+Q\rangle }_{T}=\frac{\hbar \omega }{2}n,\;E_{n-1}<E\leq E_{n},\;n=1,\;2,\;\cdots .\;$$

The values of ${\langle E_{k}\rangle }_{T}$ and ${\langle Q\rangle }_{T}$ have a similar distribution. It is noted that the wavefunction $\psi_{E}\left(x\right)$ solved from Equation (8) with an energy E in the interval $E_{n-1}<E\leq E_{n}$ is generally NSI. Our next task is to clarify the roles of these NSI wavefunctions in the quantization process of ${\langle E_{k}\rangle }_{T}$ and ${\langle Q\rangle }_{T}$.

## 5. Energy Quantization beyond SI Wavefunctions

To elucidate how SI and NSI wavefunctions cooperate to form the observed quantization levels, we consider the typical quantum motion under a quadratic confining potential $V\left(x\right)=x^{2}/2$. The related Schrödinger equation in dimensionless form is:

(23) $$\frac{d^{2}\psi_{E}}{dx^{2}}+\left(2E-x^{2}\right)\psi_{E}=0,$$

where the total energy E is allowed to be any positive real number. A general solution to the Schrödinger Equation (23), which takes into account NSI wavefunctions, can be expressed in terms of the Whittaker function W(k,m,z) as:

(24a) $$\psi_{E}\left(x\right)=\frac{C_{1}}{\sqrt{x}}W\left(\frac{E}{2},\frac{1}{4},x^{2}\right)$$

(24b) $$=C_{1}e^{-x^{2}/2}\left[F\left(\frac{1}{4}-\frac{E}{2},\frac{1}{2},x^{2}\right)/\Gamma \left(\frac{3}{4}-\frac{E}{2}\right)-2xF\left(\frac{3}{4}-\frac{E}{2},\frac{3}{2},x^{2}\right)/\Gamma \left(\frac{1}{4}-\frac{E}{2}\right)\;\right],$$

where F(α,β,z) is the hypergeometric function, and Γ(α) is the Gamma function. Detailed discussions on the above-mentioned special functions can be found in standard textbooks of physical mathematics [30]. For a given energy E, the obtained solution $\psi_{E}\left(x\right)$ is generally NSI, except for the energy eigenvalues $E_{n}=n+1/2,\;n=0,\;1,\;2,\;\cdots$, at which Equation (24b) becomes:

(25) $$\psi_{n}\left(x\right)=C_{1}e^{-x^{2}/2}\left[F\left(-\frac{n}{2},\frac{1}{2},x^{2}\right)/\Gamma \left(\frac{1}{2}-\frac{n}{2}\right)-2xF\left(\frac{1}{2}-\frac{n}{2},\frac{3}{2},x^{2}\right)/\Gamma \left(-\frac{n}{2}\right)\right].$$

Depending on whether n is even or odd, simplification of $\psi_{n}\left(x\right)$ is given respectively by:

- n=2m:
  (26a) $$\psi_{m}\left(x\right)=C_{1}e^{-x^{2}/2}F\left(-m,1/2,x^{2}\right)/\Gamma \left(1/2-m\right)=C_{1}e^{-x^{2}/2}H_{2m}\left(x\right).$$
- n=2m+1:
  (26b) $$\psi_{m}\left(x\right)=C_{1}e^{-x^{2}/2}F\left(-m,3/2,x^{2}\right)/\Gamma \left(-1/2-m\right)=C_{1}e^{-x^{2}/2}H_{2m+1}\left(x\right).$$
  where we note 1/Γ(−m)=0 in Equation (25) for negative integer −m. Combining the above two equations yields the eigenfunctions $\psi_{n}\left(x\right)=C_{1}e^{-x^{2}/2}H_{n}\left(x\right)$ for the quantum harmonic oscillator. The eigenfunctions $\psi_{n}\left(x\right)$ are the only solutions to the Schrödinger Equation (23), satisfying the boundary condition (9) and the SI condition (10).

All the existing discussions on energy quantization in the harmonic oscillator focus on the SI eigenfunctions and their linear combinations. Here we are interested in the energy quantization related to the NSI wavefunctions described by Equation (24) with E≠n+1/2. According to Equations (17) and (21), the quantization of ${\langle E_{k}\rangle }_{T}$ and ${\langle E_{k}+Q\rangle }_{T}$ is determined by the numbers of zero and pole of $\psi_{E}\left(x\right)$ and $\psi_{E}^{\prime }\left(x\right)$. Examining the expression for $\psi_{E}\left(x\right)$ given by Equation (24b), we find that $\psi_{E}\left(x\right)$ and $\psi_{E}^{\prime }\left(x\right)$ do not have any pole over the entire complex plane, because the hypergeometric function F(α,β,z) and its derivative are analytic functions for any z∈ℂ. Accordingly, we have $P_{\psi }=P_{\psi \prime }=0$, and:

(27) $$n_{\psi }=Z_{\psi }-P_{\psi }=Z_{\psi },\;n_{\psi^{\prime }}=Z_{\psi^{\prime }}-P_{\psi^{\prime }}=Z_{\psi^{\prime }},\;n_{p}=Z_{p}-P_{p}=Z_{\psi^{\prime }}-Z_{\psi }.$$

Hence the three quantum numbers, $n_{\psi }$, $n_{p}$ and $n_{\psi^{\prime }}$, can be determined by the two independent integers: $Z_{\psi }$ and $Z_{\psi^{\prime }}$, the numbers of zero of $\psi_{E}\left(x\right)$ and $\psi_{E}^{\prime }\left(x\right)$, respectively. Regarding the computation of $Z_{\psi }$, we can find the zero of $\psi_{E}$ by solving the roots of the Whittaker function according to Equation (24a):

(28) $$W\left(\frac{E}{2},\frac{1}{4},x^{2}\right)=0,\;x\in \mathbb{C},\;E\in {\mathbb{R}}^{+}.$$

**Figure 1:**
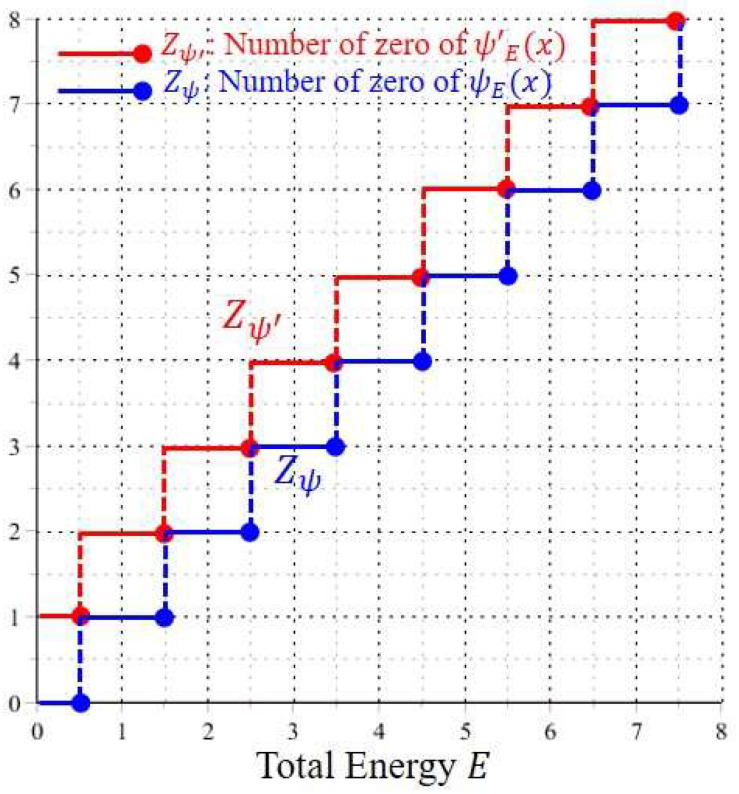
The stair-like distributions of the numbers of zero of $\psi_{E}\left(x\right)$ and $\psi_{E}^{\prime }\left(x\right)$, as the total energy E changes continuously

For a given energy E, the resulting root is denoted by $x_{s}\left(E\right)$, and $Z_{\psi }$ is the number of $x_{s}\left(E\right)$ satisfying Equation (28). The blue line in Figure 1 illustrates the variation of $Z_{\psi }$ with respect to the energy E. Similarly, $Z_{\psi^{\prime }}$ can be found by solving the roots of $\psi_{E}^{\prime }\left(x\right)=0$:

(29) $$\left(2E+1-2x^{2}\right)\cdot W\left(\frac{E}{2},\frac{1}{4},x^{2}\right)+4\cdot W\left(\frac{E}{2}+1,\frac{1}{4},x^{2}\right)=0,\;x\in \mathbb{C},\;E\in {\mathbb{R}}^{+}.$$

The resulting root is denoted by $x_{eq}\left(E\right)$ and the number of $x_{eq}\left(E\right)$ satisfying Equation (29) for a given energy E gives the value of $Z_{\psi^{\prime }}$. The red line in Figure 1 illustrates the variation of $Z_{\psi^{\prime }}$ with respect to the energy E.

**Figure 2:**
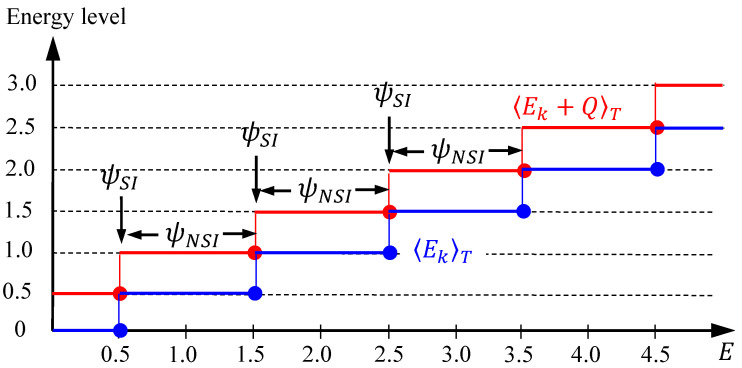
The step changes of ${\langle E_{k}\rangle }_{T}$ and ${\langle E_{k}+Q\rangle }_{T}$ occur at the SI wavefunctions $\psi_{E}$ with E=n+1/2, as the total energy E changes continuously in a harmonic oscillator. The flat parts of ${\langle E_{k}\rangle }_{T}$ and ${\langle E_{k}+Q\rangle }_{T}$ are constituted by the NSI $\psi_{E}$ with E≠n+1/2.

As can be seen from Figure 1, when the total energy E increases monotonically, $Z_{\psi^{\prime }}$ and $Z_{\psi }$ exhibit a stair-like distribution in the form of:

(30) $$Z_{\psi^{\prime }}=n_{\psi^{\prime }}=n+1,\;Z_{\psi }=n_{\psi }=n,\;n-\frac{1}{2}<E\leq n+\frac{1}{2},\;n=1,\;2,\;\cdots .$$

and $Z_{\psi^{\prime }}=1$, $Z_{\psi }=0$, as 0<E≤1/2. Based on the above distributions of $Z_{\psi^{\prime }}$ and $Z_{\psi }$, the quantization laws derived in Equations (17), (19) and (21) now become:

(31) $${\langle E_{k}\rangle }_{T}=\frac{n_{\psi }}{2}=\frac{n}{2},\;{\langle E_{k}+Q\rangle }_{T}=\frac{n_{\psi \prime }}{2}=\frac{n+1}{2},\;{\langle Q\rangle }_{T}=\frac{1}{2}\left(n_{\psi \prime }-n_{\psi }\right)=\frac{1}{2},$$

when the total energy E falls in the interval n−1/2<E≤n+1/2. All the energies in Equation (31) have been expressed in terms of the multiples of ℏω. Consequently, as we increase the total energy E monotonically, ${\langle E_{k}\rangle }_{T}$ and ${\langle E_{k}+Q\rangle }_{T}$ increase in a stair-like manner with the step levels given by Equation (31), as shown in Figure 2. Up to this stage, the two components $E_{k}$ and Q in the energy conservation law (7) have been found to be quantized, while the third component, i.e., the externally applied potential V(x), is not a quantized quantity, which otherwise changes continuously with E via the relation:

(32) $${\langle V\left(x\right)\rangle }_{T}=E-E_{k}+Q_{T}=E-\frac{n+1}{2}.$$

The most noticeable point is that the step change of ${\langle E_{k}\rangle }_{T}$ and ${\langle E_{k}+Q\rangle }_{T}$ occurs at the specific energies $E_{n}=n+1/2$, which coincide with the energy eigenvalues of the harmonic oscillator. In other words, the role of the SI condition amounts to determining the discrete energy $E_{n}$ at which the numbers of zero of $\psi_{E}\left(x\right)$ and $\psi_{E}^{\prime }\left(x\right)$ exhibit a step jump, while the role of the NSI wavefunctions $\psi_{E}\left(x\right)$ with $E\neq E_{n}$ is to form the flat parts of the stair-like distribution as shown in Figure 1 and Figure 2, where the numbers of zero of $\psi_{E}\left(x\right)$ and $\psi_{E}^{\prime }\left(x\right)$, or equivalently the time-average energies ${\langle E_{k}\rangle }_{T}$ and ${\langle E_{k}+Q\rangle }_{T}$, keep unchanged.

## 6. Quantum Bifurcation beyond SI Wavefunctions

As the total energy E increases, the wavefunction $\psi_{E}\left(x\right)$ transits repeatedly from NSI states to a SI state, once E coincides with an energy eigenvalue $E_{n}$. In this section, we will show that the encounter with an energy eigenvalue not only causes a step jump of ${\langle E_{k}\rangle }_{T}$ and ${\langle E_{k}+Q\rangle }_{T}$, but also causes a nonlinear phenomenon - quantum bifurcation, where the number of equilibrium points of the quantum dynamics experiences an instantaneous change.

With $\psi_{E}\left(x\right)$ given by Equation (24a), the quantum dynamics (11) assumes the following dimensionless form:

(33) $$\frac{dx}{dt}=-i\frac{\psi_{E}^{\prime }\left(x\right)}{\psi_{E}\left(x\right)}=\frac{i}{2x}\left(2E+1-2x^{2}\right)+\frac{2i}{x}\frac{W\left(E/2+1,1/4,\;x^{2}\right)}{W\left(E/2,1/4,\;x^{2}\right)},$$

where the total energy E is treated as a free parameter, whose critical values for the occurrence of bifurcation are to be identified. The quantum trajectories x(t) solved from Equation (33) provide us with a quantitative comparison between SI and NSI wavefunctions, which otherwise cannot be compared under the probability interpretation of $\psi_{E}\left(x\right)$.

As can be seen from Equation (33), the equilibrium point $x_{eq}$ of the quantum dynamics is equal to the zero of $\psi_{E}^{\prime }\left(x\right)$, while the singular point $x_{s}$ is just the zero of $\psi_{E}\left(x\right)$. Hence the step changes of $Z_{\psi }$ and $Z_{\psi^{\prime }}$ shown in Figure 1 also imply the step changes of the numbers of the equilibrium points $x_{eq}$ and the singular points $x_{s}$, respectively. In other words, we can say that the following two processes occur synchronously as E increases monotonically: one process is the quantization of ${\langle E_{k}\rangle }_{T}$ and ${\langle E_{k}+Q\rangle }_{T}$ regarding the step changes of $Z_{\psi }$ and $Z_{\psi^{\prime }}$ as discussed previously, and the other is the bifurcation of the quantum dynamics (33) regarding the step changes of the equilibrium points and singular points, as to be discussed below.

(1) SI wavefunctions:

Firstly, we consider the special cases that the total energy E happens to be one of the eigen energies: $E_{0}=1/2$, $E_{1}=3/2$, and $E_{2}=5/2$. The related eigenfunctions and the eigen-dynamics derived from Equation (33) are given by:

(34a) $$\psi_{0}\left(x\right)=e^{-x^{2}/2}:\;\frac{dx}{dt}=ix,$$

(34b) $$\psi_{1}\left(x\right)=2xe^{-x^{2}/2}:\;\frac{dx}{dt}=i\frac{x^{2}-1}{x},$$

(34c) $$\psi_{2}\left(x\right)=2\left(2x^{2}-1\right)e^{-x^{2}/2}:\;\frac{dx}{dt}=i\frac{x\left(x^{2}-5/2\right)}{x^{2}-1/2}.$$

These three equations describe the velocity fields and their solutions give the eigen-trajectories for the first three SI states of the harmonic oscillator. It can be shown that the equilibrium points of Equation (34) are centers, while their singular points are saddles. For instance, $\dot{x}=ix$ in Equation (34a) has an equilibrium point at the origin with solution given by $x\left(t\right)=ce^{it}$, whose trajectories on the complex plane are concentric circles around the equilibrium point, showing that x=0 is a center. To show singular points in Equation (34) are saddles, we consider the following complex-valued system with a singular point at the origin:

(35) $$\dot{x}=f\left(x\right)=\frac{g\left(x\right)}{x},\;x\in \mathbb{C},$$

where g(x) is analytic at x=0. In a neighborhood of the origin, Equation (35) can be approximated by $\dot{x}=\lambda /x$, where λ=g(0) is the residue of f(x) evaluated at x=0. The substitution of $x=x_{R}+ix_{I}$ and λ=α+iβ into $\dot{x}=\lambda /x$ leads to the equivalent real-valued nonlinear system:

(36) $$x_{R}{\dot{x}}_{R}-x_{I}{\dot{x}}_{I}=\alpha ,\;x_{R}{\dot{x}}_{I}+x_{I}{\dot{x}}_{R}=\beta .$$

**Figure 3:**
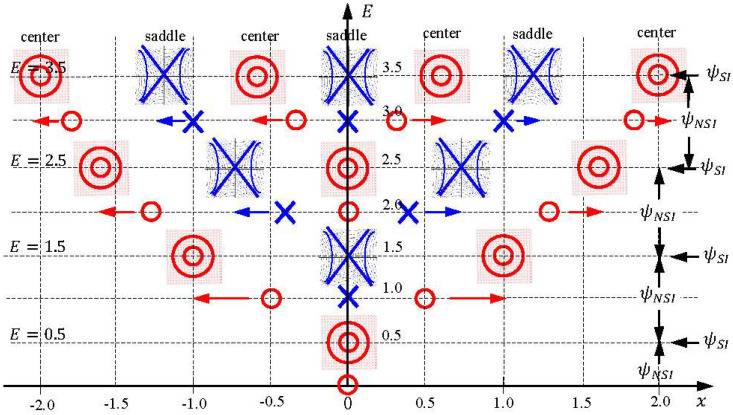
The distribution and movement of the centers and saddles over the horizontal x axis, as the total energy E changes continuously along the vertical axis.

Its solution is a set of hyperbolas expressed by $x_{R}^{2}-2\left(\alpha /\beta \right)x_{R}x_{I}-x_{I}^{2}=C$, showing that the singular point x=0 in Equation (35) are saddles. The centers and saddles of Equation (34) generated by the SI wavefunctions $\psi_{E}\left(x\right)$ with $E_{0}=1/2$, $E_{1}=3/2$, and $E_{2}=5/2$ are illustrated in Figure 3, which displays the distribution and movement of the centers and saddles of the quantum dynamics (33) on the horizontal x axis, as the total energy E changes continuously along the vertical axis. Detailed discussions on the quantum trajectories of the SI wavefunctions $\psi_{n}\left(x\right)$ for a harmonic oscillator were reported in the literature [31,32]. Here our concern is the quantum trajectories of the NSI wavefunctions $\psi_{E}\left(x\right)$ with E≠n+1/2. Quantum trajectories in the first three quantization intervals of E will be examined below, from which a global picture of center-saddle bifurcation can be drawn.

**Figure 4:**
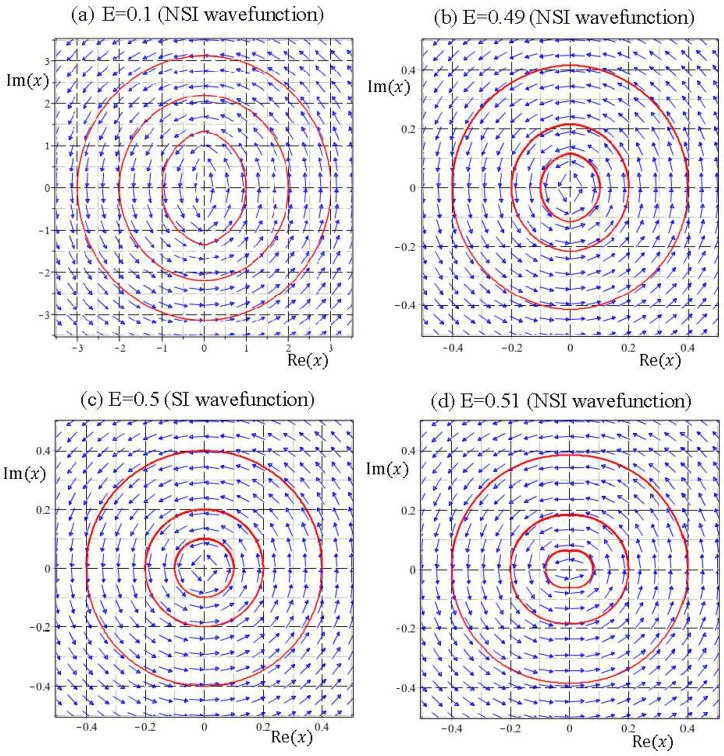
The wavefunctions corresponding to E=0.1, E=0.49, and E=0.51 are NSI, but their quantum trajectories are bound and closely connected to the eigen trajectories of E=0.5, which are concentric circles around the equilibrium point at the origin.

(2) NSI wavefunctions $\psi_{E}\left(x\right)$ with 0<E≤1/2:

The wavefunction $\psi_{E}\left(x\right)$ in this range of energy is NSI, except for E=1/2. It seems to be a reasonable conjecture that NSI wavefunctions naturally give rise to unbound quantum trajectories; however, this is not the case. Figure 4 illustrates the quantum trajectories solved from Equation (33) for the NSI wavefunctions $\psi_{E}\left(x\right)$ with E=0.1, E=0.49, and E=0.51. In spite of being generated by NSI wavefunctions, the resulting quantum trajectories are bound with slight deviations from the eigen-trajectories of E=1/2, which are concentric circles around the equilibrium point at the origin, as described by Equation (34a). It appears that SI eigenfunctions are not isolated from the neighboring NSI wavefunctions, because their quantum trajectories can be deformed continuously into each other.

**Figure 5:**
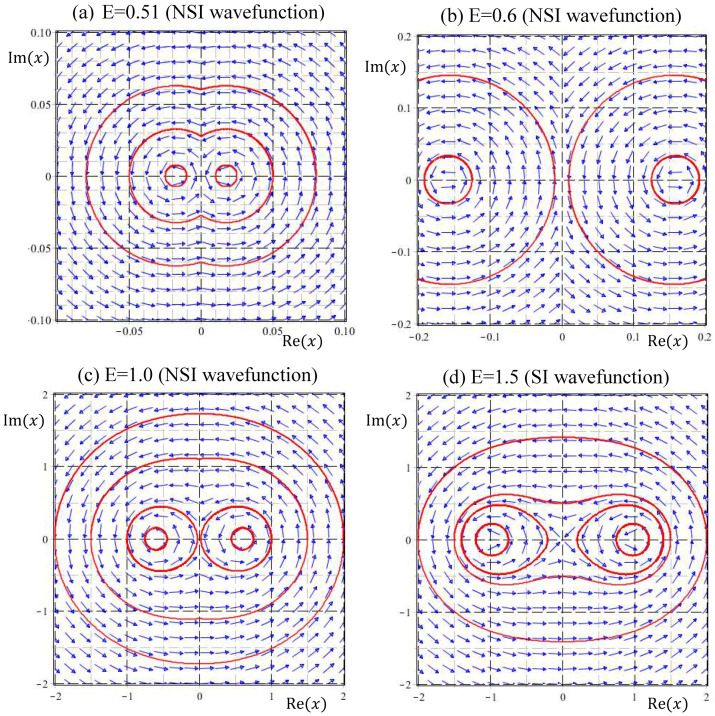
Velocity fields and quantum trajectories in the energy interval 0.5<E≤1.5 show the quantum bifurcation that the number of equilibrium points jumps from one to two as energy across E=0.5. Part (**a**) is the enlargement of Figure 4d near the origin to illustrate the split of the single equilibrium point at the origin into a pair of equilibrium points as E increases from 0.5 to 0.51. In this energy interval, there are two equilibrium points and one singular point at the origin, corresponding to the energy levels $Z_{\psi^{\prime }}=2$ and $Z_{\psi }=1$ as shown in Figure 1.

(3) NSI wavefunctions $\psi_{E}\left(x\right)$ with 1/2<E≤3/2:

According to Figure 1, the number of equilibrium points $Z_{\psi^{\prime }}$ of the quantum dynamics (33), jumps from one to two as E across the energy eigenvalue E=1/2. This bifurcation phenomenon is illustrated in Figure 5. There is only one equilibrium point at the origin in the energy interval 0<E≤0.5, while beyond the bifurcation point E=1/2, two equilibrium points come out from the origin. Particular attention is paid to the quantum trajectories of E=0.51 depicted in Figure 4d. At first glance, it looks like that the quantum trajectories of E=0.51 have a single equilibrium point at the origin. However, the enlargement of Figure 4d near the origin as illustrated in Figure 5a indicates that the single equilibrium point at the origin for E=1/2 splits into two equilibrium points as E increases to 0.51. When E increases to 3/2, the two equilibrium points (two centers) move further to $x_{eq}=\pm 1$, as described by the quantum dynamics (34b) and illustrated in Figure 5d.

Coincident with the splitting of the equilibrium point at the bifurcation point E=1/2, a singular point emerges from the origin in the form of a saddle point. The resulting saddle point pattern in the vicinity of the origin is clearly manifested in Figure 5. It turns out that at the bifurcation energy E=1/2, two kinds of bifurcation occur simultaneously: one bifurcation regards the change of the number of equilibrium points from a single center at the origin into a pair of centers moving apart along the positive and negative real axis as E increases from 1/2 to 3/2, and the other bifurcation regards the change from a center into a saddle at the origin.

**Figure 6:**
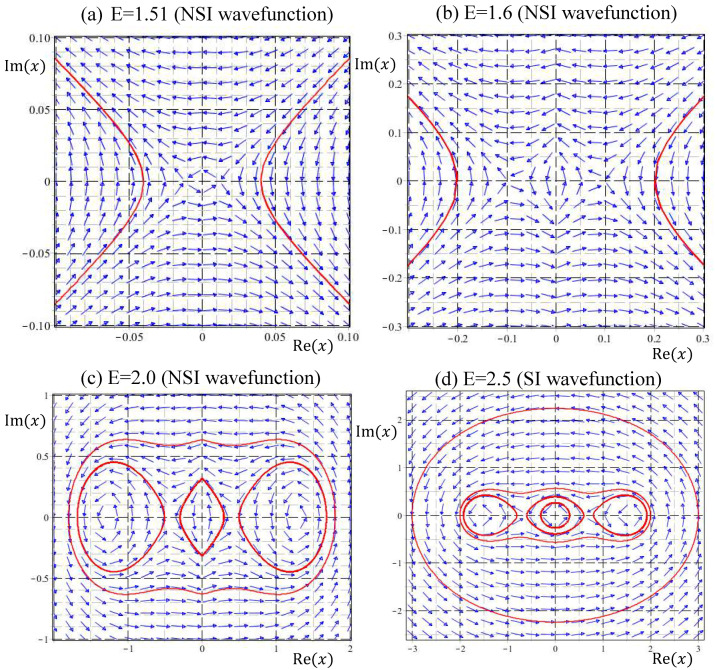
Velocity fields and quantum trajectories in the energy interval 1.5<E≤2.5 show the quantum bifurcation that the number of equilibrium points jumps from two to three as energy across E=1.5. Part (**a**,**b**) are the enlargements of the velocity field near the origin to illustrate the emergence of a new equilibrium point (a center). In this energy interval, there are three equilibrium points and two singular points, corresponding to the energy levels $Z_{\psi^{\prime }}=3$ and $Z_{\psi }=2$ as shown in Figure 1. Part (**d**) plots the eigen trajectories for E=2.5 to show the three equilibrium points at $x_{eq}=0,\;\pm \sqrt{5/2}$ and two singular points at $x_{s}=\pm \sqrt{1/2}$.

(4) NSI wavefunctions $\psi_{E}\left(x\right)$ with 3/2<E≤5/2:

At the energy eigenvalue E=3/2, the value of $Z_{\psi^{\prime }}$ experiences the second step jump, and a new bifurcation is expected to form here. This prediction is confirmed in Figure 6a, where the enlargement of the velocity field near the origin shows that the saddle-point singularity at the origin for E=3/2 now transforms into a center for E=1.51. As E increases further to E=1.6 and E=2, flow circulation around the origin as a center becomes more apparent. Counting the new equilibrium point emerging from the origin and the already existing pair of centers, the number of equilibrium points increases from two to three as E across E=3/2, and remains three in the interval 3/2<E≤5/2. Coincident with the emergence of a new center from the origin at E=3/2, the singular saddle point previously residing at the origin now splits into a pair of saddles with their separation increasing with E. The two saddles move to $x_{s}=\pm \sqrt{1/2}$ when E increases to 5/2, as described by Equation (34c) and illustrated in Figure 6d.

(5) Center-Saddle Bifurcation

**Figure 7:**
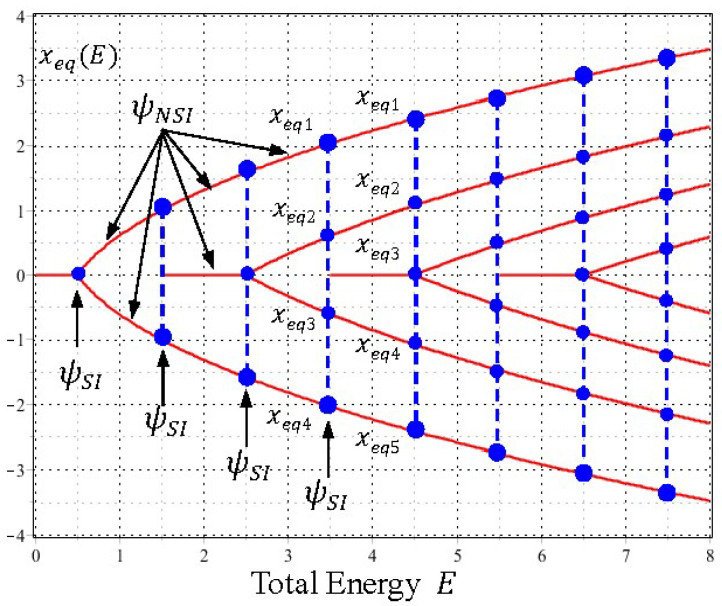
A sequence of pitchfork bifurcation curves shows the variation of equilibrium points $x_{eq}\left(E\right)$ with respect to the total energy E. The number of $x_{eq}\left(E\right)$ at each E, denoted by the blue dots, is equal to the energy level $Z_{\psi^{\prime }}$ as plotted in Figure 1. The branches of the $x_{eq}\left(E\right)$ curves start sequentially at E=0, 3/2, 7/2,⋯, and bifurcate sequentially at E=1/2, 5/2, 9/2,⋯. Except for the bifurcation points (the blue dots), the entire sequential bifurcation diagram is formed by the NSI wavefunctions $\psi_{E}\left(x\right)$ with E≠n+1/2.

**Figure 8:**
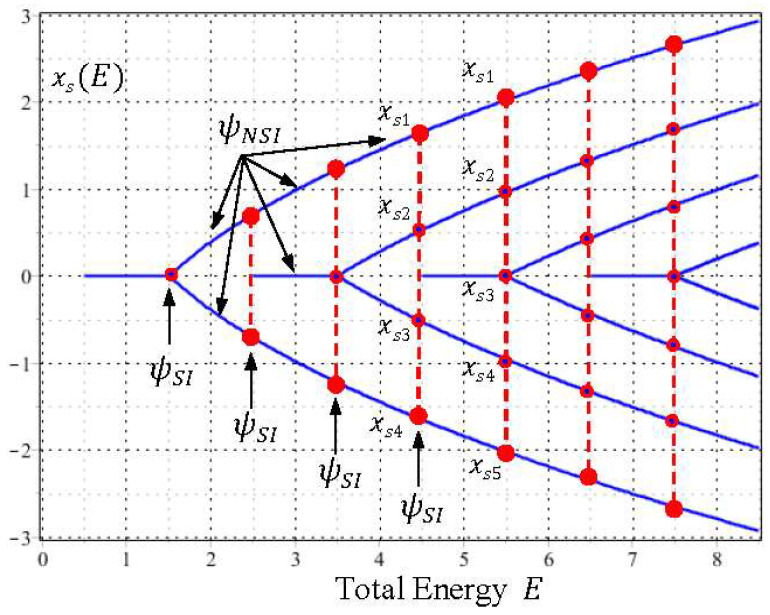
A sequence of pitchfork bifurcation curves shows the variation of singular points $x_{s}\left(E\right)$ with respect to the total energy E. The number of $x_{s}\left(E\right)$ at each E, denoted by the red dots, is equal to the energy level $Z_{\psi }$ as plotted in Figure 1. The branches of the $x_{s}\left(E\right)$ curves start sequentially at E=1/2, 5/2, 9/2,⋯, and bifurcate sequentially at E=3/2, 7/2, 11/2,⋯. Except for the bifurcation points (the red dots), the entire sequential bifurcation diagram is formed by the NSI wavefunctions $\psi_{E}\left(x\right)$ with E≠n+1/2.

When we proceed further, the bifurcations of the equilibrium centers $x_{eq}$ and the singular saddles $x_{s}$ of the quantum dynamics (33) occur alternatively as E increases. To gain a global picture of the bifurcation pattern, we solve the equilibrium points $x_{eq}\left(E\right)$ and the singular points $x_{s}\left(E\right)$ from Equations (29) and (28), respectively, and then plot them as functions of E. The resulting plots generate two sequences of pitchfork bifurcation diagram as shown in Figure 7 and Figure 8 for $x_{eq}\left(E\right)$ and $x_{s}\left(E\right)$, respectively. It can be seen that the bifurcations of $x_{eq}\left(E\right)$ and $x_{s}\left(E\right)$ occur alternatively at the critical energies $E_{n}=n+1/2$ in such a way that the branches of $x_{eq}\left(E\right)$ bifurcate sequentially at E=0+(1/2), 2+(1/2), 4+(1/2), ⋯, while the branches of $x_{s}\left(E\right)$ bifurcate sequentially at E=1+(1/2), 3+(1/2), 5+(1/2), ⋯.

Furthermore, it is worth noting that except for the bifurcation points (the blue dots in Figure 7 and the red dots in Figure 8), the sequential bifurcation diagram is constructed entirely by the NSI wavefunctions $\psi_{E}\left(x\right)$ with E≠n+1/2. Without the participation of the NSI wavefunctions, adjacent eigenfunctions lose their interconnection and a continuous description of the bifurcation sequence becomes impossible. The other perspective of center-saddle bifurcation can be gained from Figure 3, where we can see that centers and saddles appear alternatively at the origin as the total energy E increases monotonically along the vertical axis.

(6) Synchronicity between quantization and bifurcation

We recall that the number of $x_{eq}\left(E\right)$ at each E is just the number of zero of $\psi_{E}^{\prime }\left(x\right)$, which gives the quantization level of ${\langle E_{k}+Q\rangle }_{T}$. This relation indicates that the bifurcation of the equilibrium center point $x_{eq}\left(E\right)$ and the quantization of ${\langle E_{k}+Q\rangle }_{T}$ occur synchronously. Similarly, because the number of $x_{s}\left(E\right)$ at each E is the number of zero of $\psi_{E}\left(x\right)$, which gives the quantization level of ${\langle E_{k}\rangle }_{T}$, the bifurcation of the singular saddle point $x_{s}\left(E\right)$ is thus synchronous with the quantization of ${\langle E_{k}\rangle }_{T}$. Table 1 lists the numbers of saddles and centers, and the energy levels of ${\langle E_{k}+Q\rangle }_{T}$ and ${\langle E_{k}\rangle }_{T}$ for the first several energy intervals. As can be seen, the numbers of saddles and centers change synchronously with the change of ${\langle E_{k}+Q\rangle }_{T}$ and ${\langle E_{k}\rangle }_{T}$. It is noted that as energy E varies continuously during the quantization and bifurcation processes, the instantaneous changes of energy levels and equilibrium points are triggered by the SI condition E=n+1/2.

## 7. Spin Degree of Freedom beyond SI Wavefunctions

The role of the Schrödinger equation has long been considered as describing spinless particles only, because the Schrödinger charge current for the s-states of a hydrogen-like atom vanishes and produces no intrinsic angular momentum. However, based on the observation that in the absence of a magnetic field, the Pauli equation reduces to the Schrödinger equation, it was pointed out [33] that the Schrödinger equation must be regarded as describing an electron in an eigenstate of spin and not, as universally supposed, an electron without spin. According to the dBB trajectory approach [34,35], spin is interpreted as a dynamical property of electron motion and is attributed to a circulating movement of a point, i.e., to a pure orbital motion, but not to an extended spinning object. To be consistent with the Dirac theory and with the condition of Lorentz invariance, Holland [34] proposed that the Schrödinger charge current must be supplemented by a spin magnetization current, which is generated by a circulating flow of energy in the wave field of the electron [36,37].

The NSI solutions to the Schrödinger equation considered in the present paper might give an alternative explanation for the origin of particle’s spin motion. The Schrödinger Equation (8) with given energy E actually has two independent solutions. It is surprising to find that the quantum trajectories generated by the two independent solutions are indistinguishable, except for their directions of rotation. Inspecting the quantum trajectories shown in Figure 4, Figure 5 and Figure 6, it appears that all the trajectories, either generated by SI or NSI wavefunctions, rotate counterclockwise (CCW). In fact, all the trajectories produced by the general wavefunctions given by Equation (24) rotate in the same direction, because Equation (24) only gives one of the independent solutions. A complete general solution to the Schrödinger Equation (23) comprises two independent parts:

(37) $$\psi_{E}\left(x\right)=\psi_{CCW}\left(x\right)+\psi_{CW}\left(x\right)=\frac{C_{1}}{\sqrt{x}}W\left(\frac{E}{2},\frac{1}{4},x^{2}\right)+\frac{C_{2}}{\sqrt{x}}W\left(-\frac{E}{2},\frac{1}{4},-x^{2}\right),$$

where $\psi_{CCW}\left(x\right)$ is the solution considered previously and $\psi_{CW}\left(x\right)$ is the other independent solution, whose quantum trajectories rotate clockwise (CW). The wavefunction $\psi_{CW}\left(x\right)$ represents the second half of solutions to the Schrödinger Equation (23), which is NSI for any energy E and we usually take the neglect of it as granted.

**Figure 9:**
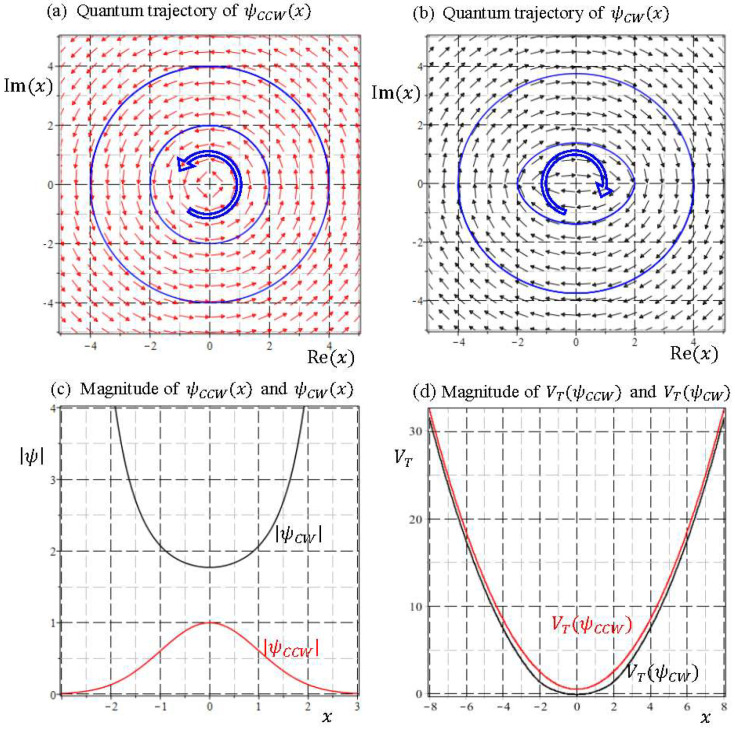
The dual-rotation solutions to the Schrödinger equation with E=1/2: (**a**) quantum trajectory of the wavefunction $\psi_{CCW}\left(x\right)$; (**b**) quantum trajectory of the wavefunction $\psi_{CW}\left(x\right)$; (**c**) comparison between $\left|\psi_{CCW}\right|$ and $\left|\psi_{CW}\right|$, and (**d**) comparison between total potential $V_{T}\left(\psi_{CCW}\right)$ and $V_{T}\left(\psi_{CW}\right)$.

The consideration of the wavefunctions $\psi_{CW}\left(x\right)$ helps to identify the additional degree of freedom independent of the particle’s orbital motion. To highlight the difference between $\psi_{CCW}\left(x\right)$ and $\psi_{CW}\left(x\right)$, their velocity fields computed by Equation (11) with E=1/2 are illustrated in Figure 9a,b, respectively. The velocity field of $\psi_{CCW}\left(x\right)$ is identical to Figure 4c, which shows circular flows surrounding the origin counterclockwise. By contrast, the velocity field of $\psi_{CW}\left(x\right)$ depicted in Figure 9b appears to be clockwise circulation around the origin. The quantum trajectories generated by $\psi_{CW}\left(x\right)$ are almost indistinguishable from those generated by $\psi_{CCW}\left(x\right)$, and the only difference between them is the directions of rotation. In addition to the orbital motion, the new degree of freedom manifested in the combination of $\psi_{CCW}\left(x\right)$ and $\psi_{CW}\left(x\right)$ is the dual directions of rotation, which is otherwise invisible along a single trajectory generated by either $\psi_{CCW}\left(x\right)$ or $\psi_{CW}\left(x\right)$. Due to their same spatial motion with dual directions, $\psi_{CCW}\left(x\right)$ and $\psi_{CW}\left(x\right)$ can be reasonably recognized as the same spatial solution to the Schrödinger equation but with opposing directions of spin.

The reason underlying the opposite rotation of $\psi_{CCW}\left(x\right)$ and $\psi_{CW}\left(x\right)$ can be explained by using the asymptotic expansion property for the Whittaker function:

(38) $$W\left(\pm k,m,\pm z\right)=e^{\mp z/2}{\left(\pm z\right)}^{\pm k}\left[1+O\left(z^{-1}\right)\right].$$

With this property, the asymptotic expansions of $\psi_{CCW}\left(x\right)$ and $\psi_{CW}\left(x\right)$ take the following forms:

(39a) $$\psi_{CCW}\left(x\right)=\frac{C_{1}}{\sqrt{x}}W\left(\frac{E}{2},\frac{1}{4},x^{2}\right)\approx C_{1}e^{-x^{2}/2}x^{E-1/2},$$

(39b) $$\psi_{CW}\left(x\right)=\frac{C_{2}}{\sqrt{x}}W\left(-\frac{E}{2},\frac{1}{4},-x^{2}\right)\approx C_{2}e^{x^{2}/2}x^{-E-1/2}.$$

The asymptotic quantum dynamics of $\psi_{CCW}\left(x\right)$ and $\psi_{CW}\left(x\right)$ then can be derived as:

(40a) $$\frac{dx}{dt}=-i\frac{d}{dx}\ln\psi_{CCW}\left(x\right)=ix-i\frac{E-1/2}{x}\approx ix,\;\left|x\right|\gg 0,$$

(40b) $$\frac{dx}{dt}=-i\frac{d}{dx}\ln\psi_{CW}\left(x\right)=-ix+i\frac{E+1/2}{x}\approx -ix,\;\left|x\right|\gg 0.$$

Irrespective to the energy E, both of the asymptotic quantum dynamics converge to the ground-state quantum dynamics (34a) with the only difference in their rotation direction. This similarity in the velocity field of $\psi_{CCW}\left(x\right)$ and $\psi_{CW}\left(x\right)$ has been ignored in the literature. Probability interpretation is only concerned with the square-integrable condition of $\psi_{CCW}\left(x\right)$ and $\psi_{CW}\left(x\right)$, as shown in Figure 9c, which obviously fails to explain the similarity in the quantum dynamics of $\psi_{CCW}\left(x\right)$ and $\psi_{CW}\left(x\right)$.

The factor dominating the similarity in the quantum dynamics of $\psi_{CCW}\left(x\right)$ and $\psi_{CW}\left(x\right)$ can be explained by Equation (5b):

(41) $$\frac{dp}{dt}=-\frac{\partial }{\partial x}V_{T}.\;$$

The total potential $V_{T}=V+Q$, comprising the applied potential V and the quantum potential Q, dominates the time evolution of the QMF p. For the case of a harmonic oscillator, $V_{T}$ turns out to be (in dimensionless form):

(42) $$V_{T}\left(\psi \right)=\frac{1}{2}x^{2}-\frac{1}{2}\frac{d^{2}}{dx^{2}}\ln\psi \left(x\right).$$

**Figure 10:**
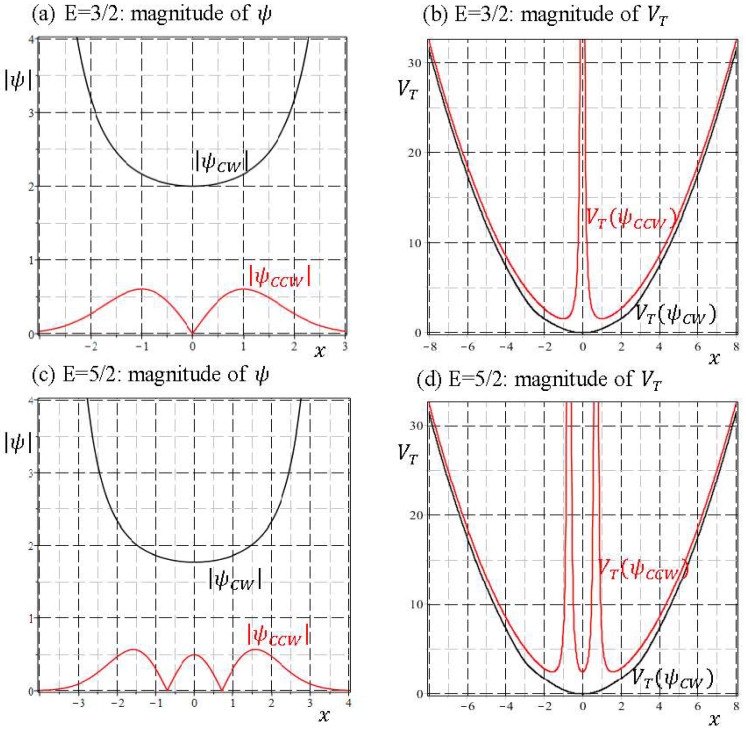
The comparisons between CCW solution $\psi_{CCW}\left(x\right)$ and CW solution $\psi_{CW}\left(x\right)$ regarding the magnitudes of ψ and the total potential $V_{T}\left(\psi \right)$ for E=3/2 and E=5/2, respectively.

The evaluations of $V_{T}\left(\psi \right)$ at $\psi =\psi_{CCW}$ and $\psi =\psi_{CW}$ for E=1/2 are plotted in Figure 9d. Despite of the adverse nature between SI wavefunction $\psi_{CCW}\left(x\right)$ and NSI wavefunction $\psi_{CW}\left(x\right)$, their total potentials exhibit a high degree of resemblance, which explains the observed similarity in the velocity fields of Figure 9a,b. The comparisons regarding the magnitudes of ψ and the total potential $V_{T}\left(\psi \right)$ for E=3/2 and E=5/2 are shown in Figure 10, where we observe that except for the region neighboring the origin, $V_{T}\left(\psi_{CCW}\right)$ is close to $V_{T}\left(\psi_{CW}\right)$ and they become identical as |x|→∞. This asymptotic identity leads to the dual velocity fields derived in Equation (40).

Schrödinger equation is a second-order differential equation with respect to its spatial coordinates, and its complete solution should be composed of two independent solutions. The common belief that Schrödinger equation is unable to describe spin motion seems to stem from our disregard of one of the independent solution. While probability interpretation of wavefunctions excludes the NSI solution $\psi_{CW}\left(x\right)$ from the general solution $\psi_{E}\left(x\right)$, the spin degree of freedom is removed at the same time. Under the framework of quantum H-J formalism, we have seen that by incorporating $\psi_{CW}\left(x\right)$ with $\psi_{CCW}\left(x\right)$ to form a general wavefunction as expressed by Equation (37), both spatial and spin motion can be described by the Schrödinger equation, and even for one-dimensional quantum motion, the spin degree of freedom can be manifested as the dual rotations on the complex x plane.

## 8. Conclusions

Standard approach to energy quantization in confined quantum systems is to seek for the allowable energies $E_{n}$ for which the time-independent Schrödinger equation has square-integrable solutions. The obtained energy eigenvalue $E_{n}$ is recognized as the quantization level of the system’s total energy. In this paper we have given a renewed interpretation for $E_{n}$ and considered NSI solutions $\psi_{E}\left(x\right)$ to the Schrödinger equation by releasing the SI requirement. The release of this requirement leads to several new findings as summarized in the following points:

- Universal quantization laws: The total energy $E=E_{k}+Q\left(x\right)+V\left(x\right)$ derived from the time-independent Schrödinger equation is shown to be conserved, but not quantized. Regardless of the confining potential V(x), quantization always occurs in the kinetic energy ${\langle E_{k}\rangle }_{T}$ and the quantum potential ${\langle Q\rangle }_{T}$, whose values can only change by an integer multiple of ℏω/2.
- Renewed meaning of the energy eigenvalues: The energy eigenvalues $E_{n}$ derived conventionally from the SI condition are shown to be the special energies at which the quantization levels of ${\langle E_{k}\rangle }_{T}$ and ${\langle E_{k}+Q\rangle }_{T}$ experience a step jump.
- The origin of energy quantization: Energy quantization in a confined system originates from the discrete change of the numbers of zero of $\psi_{E}\left(x\right)$ and $\psi_{E}^{\prime }\left(x\right)$, whose values determine the quantization levels of ${\langle E_{k}\rangle }_{T}$ and ${\langle E_{k}+Q\rangle }_{T}$.
- Concurrence of Quantization and bifurcation: Bifurcations of equilibrium center points and singular saddle points of the quantum dynamics are shown to be synchronous, respectively, with the quantization process of ${\langle E_{k}\rangle }_{T}$ and ${\langle E_{k}+Q\rangle }_{T}$, as the total energy E increases monotonically.
- Undivided SI and NSI wavefunctions: probability interpretation isolates SI wavefunctions from NSI wavefunctions; however, under the quantum H-J formalism, SI and NSI wavefunctions are indivisible with continuously connected velocity field and quantum trajectories.
- The role of NSI wavefunctions in energy quantization: Both SI and NSI wavefunctions contribute to the energy quantization. SI wavefunctions help to locate the bifurcation points at which ${\langle E_{k}\rangle }_{T}$ and ${\langle E_{k}+Q\rangle }_{T}$ have a step jump, while NSI wavefunctions form the flat parts of the stair-like distribution of the quantized energies.
- The role of NSI wavefunctions in spin: The second-order Schrödinger equation generally contains two independent solutions with opposite rotation on the complex plane. The inclusion of both solutions allows the Schrödinger equation to describe the spatial motion as well as the spin motion.

At the present stage of this research, we can only say that if the SI requirement is released tentatively, above information can be gained from NSI wavefunctions. We need further experimental results to support the existence of NSI wavefunctions in confined quantum systems. The result of this paper gives a clue to the design of such an experiment. Based on the SI condition, what we consider to be quantized is the particle’s total energy. On the other hand, if the SI condition is released, what to be quantized is the particle’s time-average kinetic energy. Experiments on particle’s motion in confining potentials can be performed to verify which prediction is correct.

## Figures and Tables

**Table 1:** The step changes of ${\langle E_{k}\rangle }_{T}$ and ${\langle E_{k}+Q\rangle }_{T}$ are synchronous with the changes of saddles and centers.

Quantized Items	$0<E<\frac{1}{2}$	$E=\frac{1}{2}$	$\frac{1}{2}<E<\frac{3}{2}$	$E=\frac{3}{2}$	$\frac{3}{2}<E<\frac{5}{2}$	$E=\frac{5}{2}$	$\frac{5}{2}<E<\frac{7}{2}$
Wavefunctions	NSI	SI	NSI	SI	NSI	SI	NSI
Zeros of $\psi_{E}\left(x\right)$	0	0	1	1	2	2	3
Number of saddles	0	0	1	1	2	2	3
Levels of ${\langle E_{k}\rangle }_{T}$	0	0	1/2	1/2	1	1	3/2
Zeros of $\psi_{E}^{\prime }\left(x\right)$	1	1	2	2	3	3	4
Number of centers	1	1	2	2	3	3	4
Levels of ${\langle E_{k}+Q\rangle }_{T}$	1/2	1/2	1	1	3/2	3/2	2

## References

[1] Styer D.F., Balkin M.S., Becker K.M., Burns M.R., Dudley C.E., Forth S.T., Gaumer J.S., Kramer M.A., Oertel D.C., Park L.H. (2002). Nine formulations of quantum mechanics. Am. J. Phys..

[2] Leacock R.A., Padgett M.J. (1983). Hamilton-Jacobi Theory and the Quantum Action Variable. Phys. Rev. Lett..

[3] Leacock R.A., Padgett M.J. (1983). Hamilton-Jacobi/Action-Angle Quantum Mechanics. Phys. Rev. D.

[4] Jordan P. (1926). Über kanonische Transformationen in der Quantenmechanik. Z. Phys..

[5] Dirac P.A.M. (1958). The Principles of Quantum Mechanics.

[6] Schwinger J. (1970). Quantum Kinematics and Dynamics.

[7] Yang C.D. (2006). Quantum Hamilton Mechanics: Hamilton Equations of Quantum Motion, Origin of Quantum Operators, and Proof of Quantization Axiom. Ann. Phys..

[8] Bohm D. (1952). A Suggested Interpretation of the Quantum Theory in Terms of Hidden Variables. Phys. Rev..

[9] Bohm D., Hiley B.J. (1993). The Undivided Universe.

[10] Holland P.R. (1993). The Quantum Theory of Motion.

[11] Gisin N. (2018). Why Bohmian Mechanics? One- and Two-Time Position Measurements, Bell Inequalities, Philosophy, and Physics. Entropy.

[12] Valentini A. (1991). Signal-locality, Uncertainty, and the Sub-quantum H-theorem, Part I. Phys. Lett. A.

[13] Valentini A., Westman H. (2005). Dynamical Origin of Quantum Probabilities. Proc. R. Soc. A.

[14] Colin S., Valentini A. (2014). Instability of Quantum Equilibrium in Bohm’s Dynamics. Proc. R. Soc. A.

[15] Bohm D., Vigier J.P. (1954). Model of the Causal Interpretation of Quantum Theory in Terms of a Fluid with Irregular Fluctuations. Phys. Rev..

[16] Efthymiopoulos C., Contopoulos G. (2006). Chaos in Bohmian Quantum Mechanics. J. Phys. A.

[17] Contopoulos G., Delis N., Efthymiopoulos C. (2012). Order in de Broglie-Bohm Quantum Mechanics. J. Phys. A.

[18] Tzemos C., Efthymiopoulos C., Contopoulosz G. (2018). Origin of chaos near three-dimensional quantum vortices: A general Bohmian theory. Phys. Rev. E.

[19] Borondo F., Luque A., Villanueva J., Wisniacki D.A. (2009). A dynamical systems approach to Bohmian trajectories in a 2D harmonic oscillator. J. Phys. A.

[20] Wisniacki D.A., Pujals E.R., Borondo F. (2006). Vortex interaction, chaos and quantum probabilities. Europhys. Lett..

[21] Wisniacki D.A., Pujals E.R., Borondo F. (2007). Vortex dynamics and their interactions in quantum trajectories. J. Phys. A.

[22] Wyatt R.E., Rowland B.A. (2007). Quantum Trajectories in Complex Phase Space: Multidimensional Barrier Transmission. J. Chem. Phys..

[23] Chou C.C., Sanz A.S., Miret-Artes S., Wyatt R.E. (2009). Hydrodynamic View of Wave Packet Interference: Quantum Caves. Phys. Rev. Lett..

[24] Goldfarb Y., Degani I., Tannor D.J. (2006). Bohmian Mechanics with Complex Action: A New Trajectory-based Formulation of Quantum Mechanics. J. Chem. Phys..

[25] Yang C.D., Wei C.H. (2016). Synthesizing Quantum Probability by a Single Chaotic Complex-Valued Trajectory. Int. J. Quantum Chem..

[26] Wilson W. (1915). The Quantum Theory of Radiation and Line Spectra. Philos. Mag..

[27] Yang C.D., Cheng L.L. (2013). Optimal Guidance Law in Quantum Mechanics. Ann. Phys..

[28] Bhalla R.S., Kapoor A.K., Panigrahi P.K. (1997). Energy Eigenvalues for a Class of One-Dimensional Potentials via Quantum Hamilton–Jacobi Formalism. Mod. Phys. Lett. A.

[29] Bhalla R.S., Kapoor A.K., Panigrahi P.K. (1997). Quantum Hamilton-Jacobi Formalism and the Bound State Spectra. Am. J. Phys..

[30] Arfken G.B., Weber H.J. (2005). Mathematical Methods for Physicists.

[31] John M.V. (2002). Modified De Broglie-Bohm Approach to Quantum Mechanics. Found. Phys. Lett..

[32] Yang C.D. (2006). Modeling Quantum Harmonic Oscillator in Complex Domain. Chaos Solitons Fractals.

[33] Hestenes D. (1975). Consistency in the formulation of the Dirac, Pauli, and Schrödinger theories. J. Math. Phys..

[34] Holland P.R. (2003). Implications of Lorentz covariance for the guidance equation in two-slit quantum interference. Phys. Rev. A.

[35] Colijn C., Vrscay E.R. (2003). Spin-dependent Bohm trajectories associated with an electronic transition in hydrogen. J. Phys. A.

[36] Ohanian H.C. (1986). What is spin?. Am. J. Phys..

[37] Mita K. (2000). Virtual probability current associated with the spin. Am. J. Phys..

